# Structural and biochemical insights into the V/I505T mutation found in the EIAV gp45 vaccine strain

**DOI:** 10.1186/1742-4690-11-26

**Published:** 2014-03-21

**Authors:** Jiansen Du, Xuefeng Wang, Jing Ma, Jianxin Wang, Yuyin Qin, Chunhui Zhu, Fang Liu, Yiming Shao, Jianhua Zhou, Wentao Qiao, Xinqi Liu

**Affiliations:** 1 State Key Laboratory of Medicinal Chemical Biology, College of Life Sciences, Nankai University, Tianjin 300071, China; 2 State Key Laboratory of Veterinary Biotechnology, Harbin Veterinary Research Institute, Chinese Academy of Agricultural Sciences, Harbin 150001, China; 3 State Key Laboratory for Infectious Disease Prevention and Control, and National Center for AIDS/STD Control and Prevention, Chinese Center for Disease Control and Prevention, Beijing 102206, China

**Keywords:** EIAV, gp45, Crystal structure, Stability, Vaccine strain, Heptad repeat, Pre-fusion conformation, Replication

## Abstract

**Background:**

The equine infectious anemia virus (EIAV) is a lentivirus of the Retrovirus family, which causes persistent infection in horses often characterized by recurrent episodes of high fever. It has a similar morphology and life cycle to the human immunodeficiency virus (HIV). Its transmembrane glycoprotein, gp45 (analogous to gp41 in HIV), mediates membrane fusion during the infection. However, the post-fusion conformation of EIAV gp45 has not yet been determined. EIAV is the first member of the lentiviruses for which an effective vaccine has been successfully developed. The attenuated vaccine strain, FDDV, has been produced from a pathogenic strain by a series of passages in donkey dermal cells. We have previously reported that a V/I505T mutation in gp45, in combination with other mutations in gp90, may potentially contribute to the success of the vaccine strain. To this end, we now report on our structural and biochemical studies of the gp45 protein from both wide type and vaccine strain, providing a valuable structural model for the advancement of the EIAV vaccine.

**Results:**

We resolved crystal structures of the ecto-domain of gp45 from both the wild-type EIAV and the vaccine strain FDDV. We found that the V/I505T mutation in gp45 was located in a highly conserved *d* position within the heptad repeat, which protruded into a 3-fold symmetry axis within the six-helix bundle. Our crystal structure analyses revealed a shift of a hydrophobic to hydrophilic interaction due to this specific mutation, and further biochemical and virological studies confirmed that the mutation reduced the overall stability of the six-helix bundle in post-fusion conformation. Moreover, we found that altering the temperatures drastically affected the viral infectivity.

**Conclusions:**

Our high-resolution crystal structures of gp45 exhibited high conservation between the gp45/gp41 structures of lentiviruses. In addition, a hydrophobic to hydrophilic interaction change in the EIAV vaccine strain was found to modulate the stability and thermal-sensitivity of the overall gp45 structure. Our observations suggest that lowering the stability of the six-helix bundle (post-fusion), which may stabilizes the pre-fusion conformation, might be one of the reasons of acquired dominance for FDDV in viral attenuation.

## Background

Lentiviruses infect mammals and induce various diseases characterized by persistent infection and progressively degenerative pathology. For example, the equine infectious anemia virus (EIAV) triggers EIA characterized by anemia, thrombocytopenia, edema, and other degenerative symptoms in horses; and the infection of human immunodeficiency virus (HIV) causes acquired immune deficiency syndrome (AIDS) [1-4]. For HIV infection, major advances have been achieved that help infected individuals to control the viral load, but the virus still cannot be completely eliminated from circulation in individuals [5]. Despite three decades of research and concerted efforts to develop an effective vaccine, the levels of protection seen in most HIV trials have been insufficient, except in a recent Thai trial (RV-144), which has presented encouraging prospects for mild immunity in the field of HIV vaccine development [6,7].

Lentiviruses rely on two glycoproteins, gp120/gp90 and gp41/gp45, that mediate attachment to the host cell membrane and viral entry [8-13]. In the case of HIV, after sequential binding of gp120 to its receptor (CD4) and its co-receptor (CCR5/CXCR4), the fusion peptides buried within the gp120/gp41 trimers are exposed and mediate membrane fusion between the virus and host cell [14-17]. The glycoprotein gp45 in EIAV plays a similar role by forming a hetero-dimer with glycoprotein gp90 on viral surfaces, mediating membrane fusion during viral invasion. The gp45/gp41 is somewhat sequestered from the viral surface, making it difficult for neutralizing antibodies to target [18,19]. However, its high sequence conservation and low level of glycosylation make the glycoprotein a potential target for vaccine development as a broad range of antibodies can be assessed. In past years, several antibodies specific to gp41, including the 2 F5, 4E10 and 10E8, have been successfully identified and have been shown to neutralize HIV [20-22]. Although their mechanism of action is unknown, the broad anti-HIV activity of these antibodies has led to extensive studies for the crystal structures of these antibodies and gp41 [23-28]. Previous X-ray crystallographic studies have confirmed that the thermostable sub-domain of HIV gp41 folds into a α-helical six-helix bundle, in which three NHR helices form an interior, parallel coiled-coil trimer while three CHR helices pack in an oblique, anti-parallel manner into the highly conserved deep hydrophobic grooves on the surface of the N-helical trimer [25,27,29]. The crystal structure of HIV gp41, reported by Buzon, V et al. (PDB code 2X7R), containing an FPPR (fusion peptide proximal) and MPER (membrane proximal external) region is the most complete structure to date [30]. However, the structure of EIAV gp45, which has a low sequence identity (approximately 20–25% according to BLAST) with HIV/SIV gp41, has not yet been determined.

Several achievements have been accomplished in vaccine development against virus-induced diseases [4,31,32]. Examples of lentivirus vaccines include, the equine infectious anemia virus (EIAV) and feline immunodeficiency virus (FIV) [33]. The effective EIAV vaccine was initially developed by both Chinese [34] and American scientists [35]. In our previous studies, we have found the Val/Ile505 to Thr (V/I505T) mutation in gp45 to be highly associated with the vaccine strains [36] and negatively correlated with the severity of pathogenic symptom in horses [37]. In the present study, we determined the crystal structure of both the wild-type (WT) and V/I505T mutant of gp45. The V/I505T mutation is located in the *d* position of the heptad repeat, protruding toward the central axis within the six-helix bundle, where high levels of conservation are observed for a range of lentiviruses including HIV and SIV. Along with biochemical and virological data, we discuss the potential association and involvement of this mutation within the vaccine strain.

## Results

### The crystal structure of EIAV gp45

**Figure 1:**
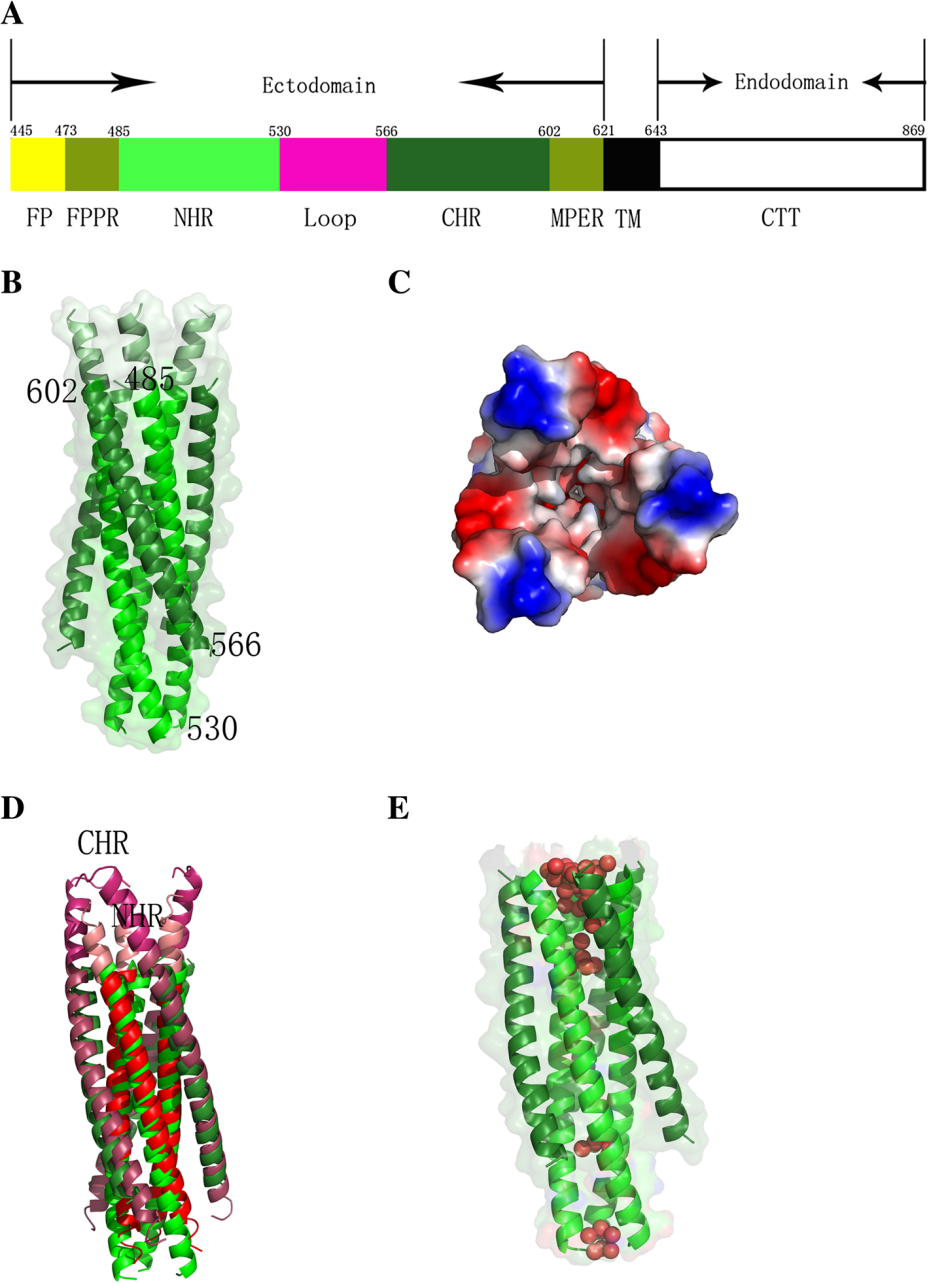
**Structural properties of the EIAV gp45 protein. (A)** Schematic representation of EIAV gp45. FP: fusion peptide; FPPR: fusion peptide proximal region; NHR: N-terminal Heptad Repeats; CHR: C-terminal Heptad Repeats; MPER: membrane proximal external region; TM: transmembrane domain; CTT: C-terminal tail. The residue numbers for demarcation of each region are shown. **(B)** Crystal structure of the wild-type (WT) protein, gp45_WT_. The full-length trimer formed through crystallographic symmetry is shown as a ribbon model. The core of the gp45_WT_ has been highlighted to represent the NHR (*green*) and CHR (*forest green*) domains, and the TEV sequence is shown in *palegreen*. **(C)** Top view of the surface charge potential of gp45 pocket. Here the negatively charged residues are colored in *red* and positively charged residues in blue. **(D)** Superimposed structures of the EIAV gp45_WT_ and the HIV gp41, including the FPPR (fusion peptide proximal, *salmon*) and MPER (membrane proximal external, *warm pink*) regions (PDB code 2X7R). The EIAV gp45_WT_ are displayed as in (B), but the TEV sequence is removed for clarity. The HIV gp41 NHR and CHR domains are highlighted in *red* and *raspberry* colors, respectively. **(E)** The water clusters (represented as *red* spheres) within EIAV gp45_WT_ trimer.

In order to study the crystal structure of EIAV gp45, we cloned the NHR and CHR regions of gp45 (strain LN40) (Figure 1A) and connected them using a five residue linker (GGSGG) [27]. The boundary of the heptad repeats was designed in accordance to the crystal structure of HIV gp41 (PDB code 2X7R) [30], which contains the longest helices reported to date in a lentivirus glycoprotein. The expressed protein was 6× His-tagged at the N-terminus, with a tobacco etch virus (TEV) protease recognition site, inserted before the gp45 sequence for the removal of the His-tag. The gp45 construct, whose sequence begins with Asp485, was expressed and purified. As expected, the overall structure of gp45 was analogous to the reported HIV and SIV gp41 (Additional file 1: Figure S1A-S1B) [25-27,29], with a Stable 6-helix bundle formed by three inner NHR and three outer CHR components (Figure 1B). However, the gp45 surface is more acidic compared to gp41, consistent with its lower calculated pI value (4.41 versus 4.92 and 5.50 in HIV and SIV, respectively) (Additional file 1: Figure S1C-S1E). Interestingly, the TEV cleavage recognition sequence (ENLYFQSNA) can be clearly traced in the electron density map, with these residues forming an extended α-helix preceding the gp45 sequence (Figure 1B). Crystal packing revealed that these additional residues were involved in interactions with neighboring molecules, which explains why the gp45 crystallization is facile, provided the 6×His-TEV sequence is retained. Despite the high similarity of the overall structure of gp45 with HIV/SIV gp41, the N-terminus of gp45, including residues derived from TEV recognition region and first five residues of gp45 (DSVQN^489^), pointed outwards and formed an open pocket at the tip of the six-helix bundle (viewed with N-terminus on top, Figure 1C). The interiors of the pocket were deep and considerably hydrophobic (Figure 1C), largely attributed to the presence of the TEV recognition sequence. For clarity, the TEV sequence was removed from the structure for further analysis.

Comparative studies of EIAV gp45 and HIV gp41, were carried out by superimposition of the gp45 onto a readily available crystal structure of HIV gp41 (PDB code 2X7R), which contained the fusion peptide proximal region (FPPR) and was the most complete HIV gp41 structure resolved to date. Remarkably, the FPPR at the N-terminus of the NHR of HIV gp41 adopts an open conformation, as in gp45, but the width of the pocket is less pronounced than that of the latter (Figure 1D). The HIV gp41 pocket appears partially opened in comparison to the completely open state of gp45. The pocket contains a cluster of ordered solvent molecules, coordinated by the residues corresponding to the glutamine-rich layer of HIV gp41 [38], i.e., Gln488, Thr491, Glu495, and Thr498 (Figure 1E). Additionally, two water clusters are formed through the coordination of Thr519 and Gln530 at the C-terminus of NHR (Figure 1E). Such clusters of solvent molecules along the central axis of the six-helix bundle are unique to gp45. In contrast, only three water molecules are found on the axis in gp41 [29], coordinated by Gln552, Gln562, and Thr569, respectively.

### Crystal structure of the 505 T mutant

**Figure 2:**
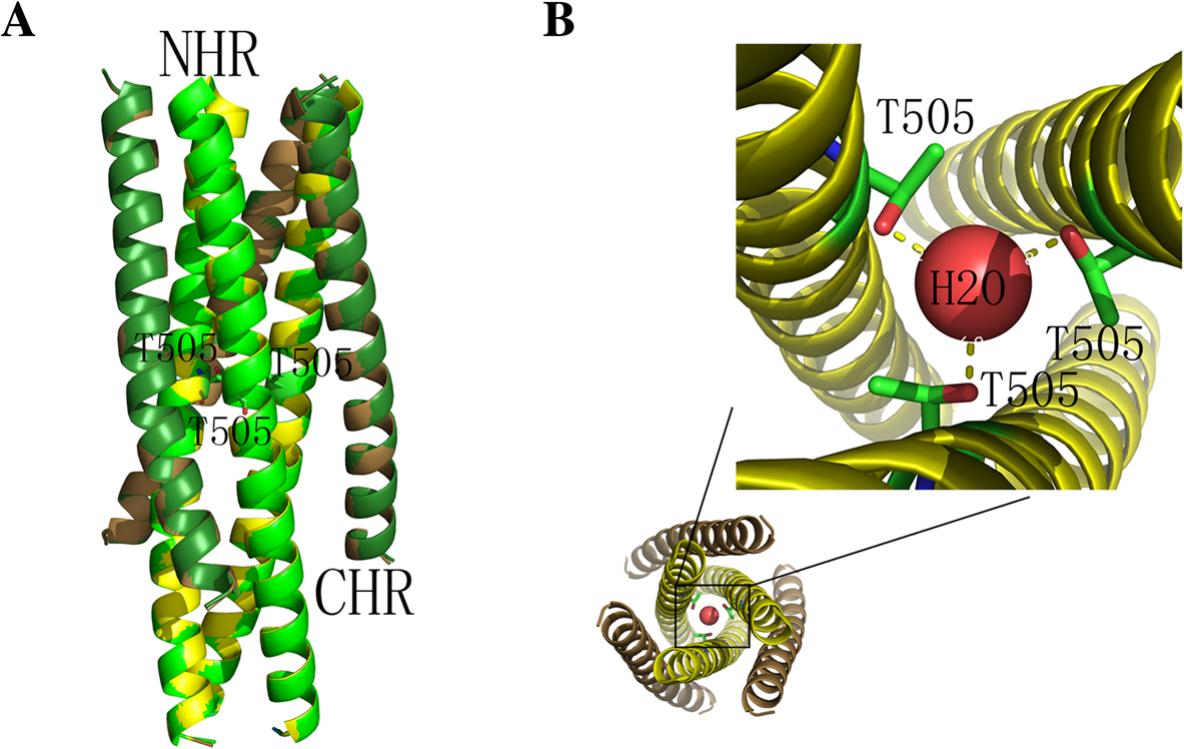
**Structural properties of the EIAV gp45**_**VACCINE**_**. (A)** Superimposed structures of EIAV gp45_WT_ and gp45_VACCINE_. gp45_WT_ are shown as in Figure 1D, and gp45_VACCINE_ is colored in *yellow* for the NHR and *sand* for the CHR. Thr505 is represented as a stick model and colored according to the element type (N, *blue*; O, *red*; C, *green*). **(B)** Thr505 in gp45_VACCINE_. The core of the gp45_VACCINE_ protein is colored as in **(A)**. Thr505 is represented as a stick model and colored according to the element type (N, *blue*; O, *red*; C, *green*), and the water molecule coordinated by the Thr505 is highlighted in *red*.

The crystal structure of gp45 derived from the vaccine strain was obtained using a procedure similar to that for the wild-type. Compared to the latter, gp45 of the vaccine strain has acquired a Val/Ile to Thr point mutation at residue 505, located at the *d* position within the heptad repeat. The crystal structure suggests that the mutation has negligible effect on the overall conformation of gp45 (Figure 2A), despite the local interaction around residue 505, which is no longer hydrophobic, but rather hydrophilic. The three hydroxyl groups of Thr coordinate a water molecule in the center (Figure 2B). Thr505 is located within neighboring hydrophilic residues corresponding to the Gln-rich region of gp41, despite the presence of an EVENN sequence (E493-N497) instead of QQQNN (HIV clade B) or QQQQQ (SIV) in EIAV gp45.

The open pocket on N-terminus of gp45 in both wide-type and 505 T mutant reminds us a conformational change might occur at this region during viral infection. Interestingly, in the recently published crystal structure of HIV gp140 Env (PDB code 4NCO), the NHR helix in gp41 is bent in the middle (Additional file 2: Figure S2)[39], indicating a huge conformational change for gp45/gp41 might occur during viral infection.

### Stability of the 6-helix bundle within EIAV gp45

**Figure 3:**
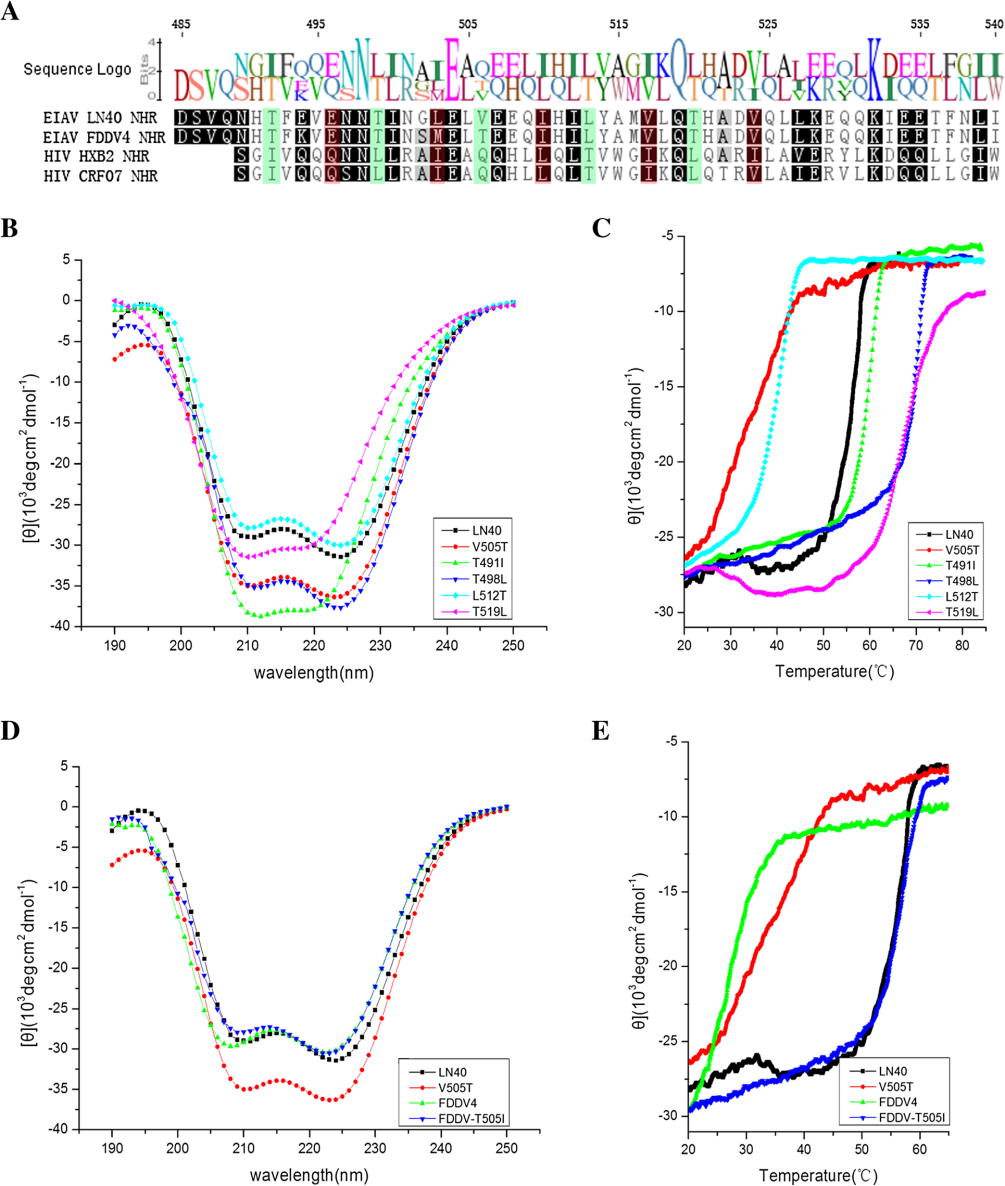
**Circular dichroism (CD) stability analyses of EIAV gp45 and its mutant proteins. (A)** The sequence alignment of the NHR region for both EIAV gp45 and HIV gp41 is shown. The mutants LN40 and FDDV4 refer to the sequences of the EIAV WT and vaccine strains, respectively. Two HIV sequences, one typical clade B (HXB2) and the other from CRF07 circulating in China, are aligned against EIAV. The *a* and *d* positions are highlighted in *orange* and *green shadows,* respectively. **(B)** Secondary structures of the EIAV gp45 protein and its mutants as characterized by CD, at room temperature. The α-helices of gp45 are well-retained across all mutants. **(C)** Temperature sensitivity measurements. Thermostability of gp45 and its mutants was monitored by CD at 222 nm, along a temperature gradient. **(D)** Secondary structures of the EIAV gp45 from the vaccine strain, FDDV4, and its mutants characterized by CD, at room temperature. **(E)** Temperature sensitivity measurements of EIAV gp45 from the vaccine strain FDDV4 and its mutants. Thermostability was monitored as described above. Results are an average of three independent experiments.

We found that the *d* positions within the heptad repeats were conserved due to their direct contribution to the stability of the helix bundle. By sequence alignment of the NHR regions of EIAV and HIV, we obtained distinct differences in the residue polarity at the *d* positions in EIAV and HIV, predominantly due to the switch between the hydrophobic Ile/Leu/Val and hydrophilic Thr (Figure 3A) residues. In the EIAV vaccine strain, the V/I505T mutation at the position 505 changes the polarity of the residue side chains. In order to determine the influence of this mutation on the *d* position, we used circular dichroism to measure protein stability under increased environmental temperatures. Compared to the WT gp45, the V/I505T mutant has similar dynamics at room temperature (Figure 3B). However, with increasing temperatures, the V/I505T mutant unfolds more rapidly than WT and has a lower thermal melting (Tm) by approximately 20°C, indicating that the stability of gp45 from the vaccine strain is markedly affected (Figure 3C). Similar mutations were tested at other *d* positions within the construct. We observed that a hydrophobic to hydrophilic mutation at Leu512 to Thr also decreased the stability of the helix bundle, albeit with less significance (Figure 3C). In contrast, the hydrophilic to hydrophobic mutations at residues 491, 498 and 519 increased the Tm due to the introduction of a hydrophobic force (Figure 3C). Within the heptad repeat, the *a/d* position point to the center and are critical for maintaining stability of the helix bundle by attaching the three NHRs together. The mutation effects at *a* position are consistent with this observation (Additional file 3: Figure S3A-S3B). For example, E495Q mutation increases the Tm significantly, possibly a result of removal of charge repulsion. Whereas conserved mutations, such as L502I, I509L, and V516I have minor effects on Tm. Due to the symmetrical packing of the three NHRs, a mutation in one molecule at the *a/d* position will introduce three corresponding mutations clustering at the same site within the helix bundle, thus significantly affecting the overall stability.

In the production of the vaccine EIAV, quasi-species harboring multiple mutations were generated during viral replication and evolution [40]. Thr505 residue exhibited increased association to the vaccine strain, although other mutations were also present in the different vaccine strain sequences studied (Additional file 3: Figure S3C). A dominant vaccine strain isolate, FDDV4, was used to make the reversion mutation to test the influence of Thr505. FDDV4 has residues S501M502 in place of G501L502 in the WT virus (Figure 3A and C). Hence, the T505I FDDV4 gp45 mutant was generated and measured by circular dichroism for its influence on protein stability. Comparable results were attained, similar to those of previous mutants (Figure 3D-E), indicating that the V/I505T mutation was crucial for gp45 stability, whereas mutations including S501M502 were negligible.

### EIAV replication of wild-type and vaccine strains

**Figure 4:**
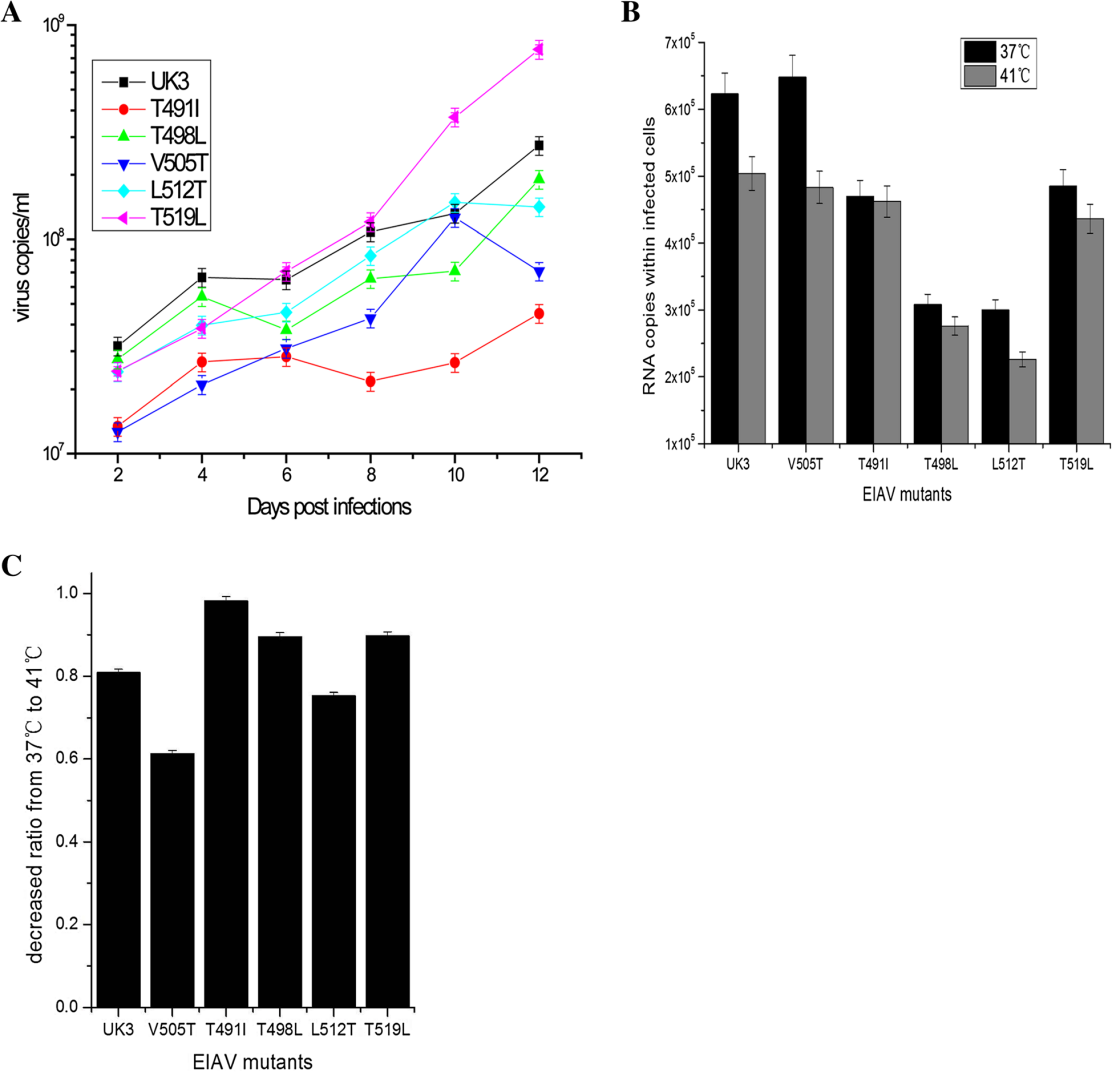
**EIAV replication kinetics and temperature sensitivity assays. (A)** Replication analysis of wild-type (WT) EIAV and EIAV with mutations at position *d*. The procedure employs ED cells which are infected by the virus in sets of three. EIAV replication kinetics is assayed using qPCR, by measuring the virions in the supernatant. **(B)** Temperature sensitivity measurements; at 41°C, each virus experiences a infection decrease compared to a temperature of 37°C. The same behavior is observed for WT but to a different degree. **(C)** The infection ratios from 37 to 41°C. The V505T mutation exhibits significant sensitivity with a considerable drop in infection as the temperature is increased. The L512T mutant is also affected in a similar manner, while the other mutants tested show minimal differences. Results are an average of three independent experiments.

Since helix bundle formation provides the energy to drive membrane fusion at the late stage of the viral entry [41], the stability of gp45 is considered critical for membrane fusion efficiency and viral infection. This is confirmed through circular dichroism, where a decrease in stability is usually accompanied by a reduction in viral infection. Our previous work shows that the *in vitro* replication ability in several cell lines of the vaccine strain is comparable to that of the WT [40]. However, our previous work is based on quasi-species composed of multiple evolutionarily-related viral sequences. To investigate the effect of mutations at the *d* position, specifically at residue 505 within the heptad repeats, we used infectious clones for EIAV [42] and introduced the corresponding mutations to determine their influence on viral replication ability in ED (Equine dermis) cells. Using this method, we were able to exclude the influence of sequence divergence in other regions outside the NHR. Although, the Thr mutation at residue 505 reduced the stability of gp45, replication ability was maintained to the level of the WT (Figure 4A). The majority of the remaining mutations at *d* also displayed tolerance and yielded viruses with replication comparable to WT, although a significant decrease was seen in the mutant T491I (Figure 4A). This observation indicated that, in spite of high conservation at the *d* position, certain mutations were tolerable for the viability of EIAV. This conclusion is also supported by the mutations in the *a* position (Additional file 4: Figure S4).

As measured by circular dichroism, we observed that the Tm of gp45 harboring a V505T mutation dropped to 38°C (close to the normal equine body temperature). However, the early stage of EIAV infection, during which the balance between the virus and host immune system is adjusted, is characterized by febrile episodes [12]. To determine whether fluctuations in body temperature play a role in virus-host interaction, we measured the viral infection ability of these mutants at varying temperatures. When the viral infection assay was performed at a higher temperature of 41°C, we observed an apparent reduction in the infection efficiency of almost all viral mutants (Figure 4B). For the virus bearing the Thr505 mutation, this reduction was significant compared to the WT (Figure 4C), indicating increased sensitivity to temperature differences. An additional mutant with reduced helix bundle stability, L512T, also displayed similar tendencies to temperature changes (Figure 4C), although the transitions were less prominent compared to Thr505. Furthermore, the mutants harboring hydrophilic to hydrophobic mutations T491I, T498L, and T519L were less sensitive to temperature in comparison with the WT (Figure 4C), possibly a result of increased stability of the helix bundle. From these mutations, a positive correlation between helix bundle stability and insensitivity to rising temperatures for virus infection is proposed, with the V505T mutant exhibiting the most distinctive change associated with temperature dependence.

## Discussion

EIAV gp45 and HIV gp41 are members of type I membrane fusion proteins, which are conserved in most RNA viruses [43]. In these proteins, the core region of the fusion machinery is composed of two heptad repeats, NHR and CHR, preceded by the fusion peptide and followed by the transmembrane region [44]. The hydrophobic fusion peptide is buried within the envelope trimer prior to fusion and is exposed during the process, leading to the transformation of NHR and CHR. This highly conserved fusion machinery provides an exceptional opportunity to study the common mechanisms underlying viral membrane fusion. For lentiviruses, useful analogies to HIV gp41 can be drawn from studies on gp45 of EIAV, notably in viral adaption. In the present study, for the first time, high-resolution crystal structures of the EIAV gp45 protein have been solved. We have carried out extensive studies on the gp45 protein derived from both the WT and vaccine strain. Additionally, a critical mutation, V/I505T, located at the highly conserved *d* position within the heptad repeat, has prompted investigations into the association of this mutation within the viral attenuation mechanism.

The membrane fusion activity of gp41 is generally proportional to its post-fusion stability [38]. Mutations that reduce stability commonly lead to a reduction of fusion ability and viral replication. In the case of gp45, a V/I505T mutation results in a significant decrease in protein stability without an accompanying decrease in viral replication. The crystal structure shows that this residue is located in the center of the post-fusion six-helix bundle, adjacent to the glutamine-rich layer. The glutamine-rich layer, characterized by a hydrophilic environment and several hydrogen bonds, is critical to the conformational transition of envelope proteins (during the infection process) [45,46]. The location of this residue is likely to be pivotal to the success of the EIAV vaccine, as no other site within gp45 has been previously associated with this specific vaccine strain. The lower stability of gp45 induced by V/I505T mutation may potentially slow down the formation of post-fusion conformation and stabilize the pre-fusion counterpart, and consequently provide a prolonged period for the antibodies to recognize exposed epitopes. Recently, stabilizing the pre-fusion conformation of the RSV F protein has proven to be an effective strategy to induce improved antibody responses in animal models [47].

The decreased stability of gp45 by V/I505T mutation might also favor the viral replication in specific cell lines, such as donkey dermal cells, a hypothesis which requires further elucidation. Our previous study identified a truncating mutant of gp45 in specific vaccine strains, which resulted in the lack of the C-terminal cytosolic region. This mutant has lower replication in monocyte-derived macrophage (MDM) but higher replication in fetal donkey dermal cell (FDD), indicating an adaptation depending on the host cell context [48]. The vaccine virus (FDDV) was adapted in 37°C by serial passing *in vitro*. In the absence of pressure from the host immune system, the V/I505T mutation in gp45 may offer an advantage for the virus in adaptation in specific cells (such as FDD) by increasing the stability of Env pre-fusion conformation, a tendency that cannot be acquired easily in an *in vivo* environment.

Our structures of EIAV gp45 also revealed differences between EIAV gp45 and HIV gp41. EIAV gp45 had a more loosely packed structure compared with HIV gp41, reflected by a higher number of coordinated water molecules at the center of the gp45 trimer and observed lower melting temperatures of the six-helix bundle. The EIAV is known to utilize the ELR1 as its sole receptor [49], in contrast to the sequential receptor/co-receptor usage of CD4 and CCR5/CXCR4 by HIV in viral entry [50,51]. Therefore, the one-step transition within EIAV suggests a simple conformational change in the EIAV envelope protein.

Additionally, the melting temperature (Tm) of gp45 is also likely to play a role in EIAV vaccine development. We found that the Tm of WT gp45 was 58°C, much lower than that of the HIV gp41 (~80°C) [29]. The gp45 of the vaccine strain, harboring the Thr505 mutation, had a Tm of only 38°C, well within the range of normal equine body temperature. This gp45 is expected to be significantly sensitive to alterations in body temperature. The EIAV vaccine strain was established by serial passing in donkey cell lines *in vitro* at 37°C, encouraging viral adaptation to this temperature but making it considerably more sensitive to temperature fluctuations during febrile episodes. This alteration may allow the host to control viral load easily, while simultaneously maintaining a continuous low level of viral replication asymptomatically and as a result driving the mutual co-evolution of the virus and its host.

In EIAV vaccine, the attenuated vaccine virus provides a high level of immunity, presumably due to the continuous antigen presentation and consistent optimization of the host immune system. Recent EIAV vaccine studies have indicated that the attenuated virus must achieve a critical level of replication to sufficiently drive the maturation of the host immune [52]. Craigo, JK et al. demonstrated that in every recurring disease cycle, a new quasi-species can appear with each fever, and the dominating isolate changes each time [53]. Therefore, it is imperative for the EIAV vaccine strain to evolve consistently *in vivo* to achieve the effective protection required by the host. Importantly, the level of immunity observed with the vaccine correlates with that of the divergence evolved from the original viral Env [35]. Hence, the studies on EIAV, including what has been reported here, provide in-depth insights for the elucidation of the vaccine mechanism, and may be helpful for other viral vaccine developments.

## Conclusions

In summary, we have carried out structural and biochemical studies of the EIAV gp45 from both wide-type and vaccine strain. A hydrophobic to hydrophilic interaction change in the EIAV vaccine strain was found to modulate the stability and thermal-sensitivity of the overall gp45 structure. Our studies suggested that the unique features of gp45, such as loose packing and low Tm, contribute to the success of FDDV in EIAV vaccine development. Our studies provide useful information on the underlying mechanism of viral adaptation during *in vitro* attenuation, and direct a strategy for further development of an effective lentiviral vaccine. Recently, the full-length gp140 structures consisting of all the variable regions have been reported, using both crystallographic and cryo-electron microscopy techniques [39,54]. These distinct structures reveal that the gp41 trimer for NHR region is pre-formed within native viral envelope, which implies that varying the heptad repeat residues at the *d* positions can have a direct impact on the stability of viral spikes, offering new implications for the design of envelope protein immunogens.

## Methods

### Plasmids and molecular cloning

For crystallography structure analysis, the EIAV gp45 was constructed by overlapping the NHR and CHR regions with a GGSGG linker. Both the NHR and CHR were amplified by polymerase chain reaction (PCR) using the gene encoding full-length template of EIAV gp140 (gp90 + gp45) and further cloned by employing the ligation-independent cloning (LIC) technique. The pET30-TEV/LIC was digested with SspI and extracted using a gel extraction kit (Axygen). Subsequently, the cleaved plasmids and purified PCR products were digested with a T4 DNA polymerase (Promega) in the presence of dGTP or dCTP, respectively. The annealed mixture containing pET30-TEV/LIC-gp45 was transformed into *E.coli* DH5α competent cells for plasmid propagation. Using this method, a 6× His-tag and a TEV cleavage site derived from the LIC vector were fused upstream of gp45. The QuikChange site-directed mutagenesis kit (Stratagene) was used to generate point mutations. All procedures were performed in accordance with the manufacturer’s instructions.

### Protein expression and purification

6× His-tagged gp45 was expressed in *E.coli* Rosetta™ (DE3) and cultured in Luria-Bertani (LB) medium, supplemented with kanamycin (50 μg/mL). The cells were grown at 37°C until an OD_600_ of 0.8 was reached. Recombinant protein expression was induced by the addition of 200 μM isopropyl β-D-1-thiogalactopyranoside (IPTG) and allowed to proceed for 12 h at 20°C. The cells were harvested by centrifugation (6000 × *g*, for 20 min, at 20°C) and re-suspended in a buffer consisting of 25 mM Tris, 500 mM NaCl, 20 mM imidazole, at pH 8.0. Subsequently, cells were lysed by sonication and the crude extract was clarified by centrifugation at 38,000 × *g*, for 40 min at 4°C.

The supernatant was applied onto a pre-equilibrated Ni–NTA resin, consisting of buffer A (25 mM Tris, 500 mM NaCl, at pH 8.0) and transferred into a column. The column was washed five times with buffer A containing 40 mM imidazole. Subsequently, proteins bound to the column were eluted with the buffer containing 500 mM imidazole. The eluent was further purified using a Q-Sepharose HiTrap HP column (GE Healthcare) with a linear concentration gradient of NaCl ranging from 100 to 500 mM. Peak fractions were collected and polished using a Superdex-200 gel filtration column (GE Healthcare). The purified protein was concentrated to 15 mg/mL and its purity was determined to be >95%, as analyzed by SDS-PAGE and Coomassie blue staining (data not shown).

### Crystallization

Crystals of EIAV gp45_WT_ (derived from the sequence of strain LN40) were grown using the sitting-drop vapor diffusion method at a constant temperature of 20°C; by mixing equal volumes (1:1 μL) of both the protein (15 mg/mL in 20 mM Tris, 500 mM NaCl, pH 8.0) and reservoir (0.1 M Bis-Tris, 2 M NaCl, pH 5.5) solutions. EIAV gp45_VACCINE_ crystals (gp45_WT_ with the V505T mutation) were grown at 20°C in 0.2 M NaCl, 0.1 M sodium acetate trihydrate, 30% v/v (+/-)-2-methyl-2,4-pentanediol, at pH 4.6. Following this, the gp45_WT_ crystals were immersed in 100% paraffin oil for several seconds and the gp45_VACCINE_ crystals were washed in the reservoir solution, prior to freezing in liquid nitrogen for storage.

### Data collection and structure determination

Data collection was performed at the Shanghai Synchrotron Radiation Facility. All datasets were collected at a wavelength of 0.9792 Å (at 100 K), processed using the HKL2000 package [55], and converted to amplitude using the CCP4 suite [56]. Data collection and processing results are summarized in Additional file 5: Table S1.

The structure of EIAV gp45_WT_ was solved by molecular replacement using the HIV gp41 structure (PDB code 2X7R) as a search model. Initial models were refined with Phenix [57] accompanied by several cycles of manual building using COOT [58]. Simulated annealing, positional and B-factor refinements were used in multiple rounds to improve the overall quality of the structure. Ordered water molecules were added to the structure in the last round of refinement. The structure of EIAV gp45_VACCINE_ was solved following similar procedures by using gp45_WT_ as the initial model. All models had low R and R_free_ factors, good deviations from ideal geometry and no Ramachandran outliers. The refinement statistics are summarized in Additional file 5: Table S1.

### Circular dichroism spectroscopy

The protocol was adapted from a previously published method [29,59]. Briefly, circular dichorism spectra were acquired on a Biologic M450 spectropolarimeter (BioLogic Science Inc., France) equipped with a thermoelectric temperature controller. Spectra of each protein were measured in PBS buffer, at 5°C in 1 nm increments, from 190 to 250 nm. For thermal denaturation measurements, the ellipticity was measured at 222 nm with 1°C increments from 20°C to 95°C, at a rate of 90°C/h. Thermal melting (Tm) points were calculated with a Boltzmann sigmoidal fit using OriginLab.

### EIAV and mutants production

Wild-type (WT) EIAV was generated from an infectious molecular clone. The clone EIAV UK3 [4] was generously provided by Dr. Montelaro (University of Pittsburgh). The QuikChange site-directed mutagenesis kit (Stratagene) was used to generate point mutations. Viruses were produced in ED (Equine dermis, ATCC CCL57) cells by transfection of the infectious clone using Lipofectamine 2000 (Invitrogen). The EIAV viruses were harvested from cultured supernatants at 72 h post-transfection. After centrifugation at 1,500 × *g* for 10 min, the supernatants were separated through a 0.45 μm filter and stored at -80°C. Viral titers were determined by quantitative PCR (qPCR). Quantified viruses were used for infection of the next passage of ED cells.

### RNA isolation and quantitative PCR (qPCR)

Viral RNA was isolated from a 140 μl supernatant using the Qiagen Viral RNA Mini Kit and the whole-cell RNA was isolated using Trizol reagent. In general, the monolayer cultures were rinsed with ice-cold PBS (50 mM phosphate, 150 mM NaCl, at pH 7.4) and lysed upon the addition of 100 μl Trizol in each well, when using a 96-well plate. Phase separation was performed by adding chloroform to the wells and the RNA was precipitated using isopropanol. Finally the RNA pellet was washed in 75% ethanol, air-dried, and re-dissolved in water.

The isolated RNA was reverse transcribed and amplified using reverse transcription PCR (RT-PCR) with a PrimeScriptTM RT reagent kit, gDNA Eraser (TaKaRa) and SYBR Premix Ex TaqTM (TaKaRa); SYBR standards RSq > 0.98, 95% < Eff. <105%.

### EIAV replication kinetics and temperature dependent studies

To measure the replication kinetics of EIAV, ED cells (5,000 cells/well) were infected by wild-type (UK3) and mutant EIAV viruses (5 × 10^6^ copies/well). In this case, the supernatant was harvested in 2-day intervals, up to 12 days post-infection. Based on the isolated viral RNA, the virus number was quantified to produce replication curves.

To examine the temperature dependence of EIAV and its mutants for viral entry, ED cells were incubated at 41°C for 2 h, prior to infection. Upon exposure, the cells were incubated for a further 2 h. Total RNA was then isolated from the infected cells and the number of EIAV entering the ED cells was determined using qPCR.

### Accession number

Atomic coordinates and structure factors have been deposited in to the RCSB Protein Data Bank under ID codes 3WMI and 3WMJ for gp45_WT_, gp45_VACCINE_, respectively.

## Competing interests

The authors declare that they have no competing interests.

## Authors’ contributions

XL and WQ conceived the project. FL, JZ, YS, WQ, and XL designed the experiments. JD, XW, JM, JW, YQ, CZ and XL performed the experiments. YS, WQ, and XL analyzed the data. XL and JD wrote the paper. All authors read and approved the final manuscript.

## Supplementary Material

Additional file 1: Figure S1 Supporting data for the EIAV gp45_WT_ structure. (A) Superimposed structures of EIAV gp45_WT_ (from Figure 1D) and the HIV CRF07 gp41 protein. The HIV CRF07 gp41 NHR and CHR domains are highlighted in *magenta* and *pink* colors, respectively, with a linker (*yellow*) between them. (B) Superimposed structures of EIAV gp45_WT_ (from Figure 1D) and the SIV gp41 protein. The SIV gp41 NHR and CHR domains are highlighted in *cyan* and *orange* colors, respectively. (C-E) Surface charge potentials of the EIAV gp45, HIV CRF07 gp41, and SIV gp41. Negatively charged residues are colored in *red* and positively charged residues in *blue*. Click here for file

Additional file 2: Figure S2 Superimposition of gp45_VACCINE_ NHR and HIV gp41 NHR derived from crystal structure of Env trimer (PDB code 4NCO). Side-view for (A) and top-view for (B). gp45_VACCINE_ NHR is shown as in Figure 2A and gp41 NHR is shown in blue. Click here for file

Additional file 3: Figure S3 Supporting data for the EIAV gp45 mutant proteins. (A) Secondary structure representation of the EIAV gp45 mutants at position *a*, characterized by CD. (B) Thermostability results monitored by CD (at 222 nm) for the *a* mutants. (C) Sequence alignment of the EIAV gp45 ecto-domain. The LN40 represents the WT pathogenic strain isolated in Liaoning Province, China. The DV/DLV34/DLV61 series is classed as a pathogenic strain; the DLV121/DLV137/FDDV4/FDDV13/FDDV23 as non-pathogenic; with DLV91 depicted as the intermediate. The UK3 pathogenic strain is used in this study to construct the infectious clone. The specific residue at the 505 position is shown in *green shadow.* Click here for file

Additional file 4: Figure S4 Replication analyses of the EIAV mutants at position *a* (as depicted in Figure 4A). Click here for file

Additional file 5: Table S1 X-ray crystallographic data and refinement statistics for EIAV gp45. Click here for file
