# Supplementary figures and images for: Structural and biochemical insights into the V/I505T mutation found in the EIAV gp45 vaccine strain
Source: Retrovirology. 2014 Mar 21;11:26. doi: 10.1186/1742-4690-11-26 (PMC3997929; doi:10.1186/1742-4690-11-26)

A

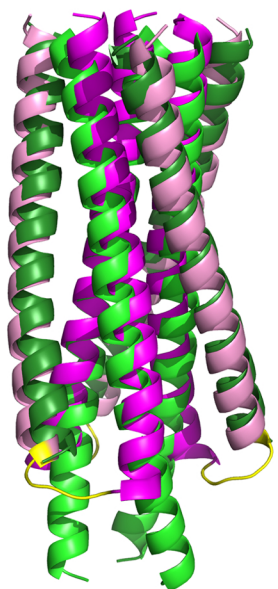

B

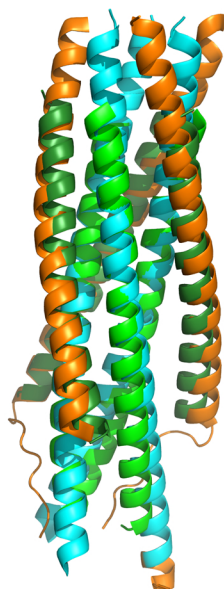

C

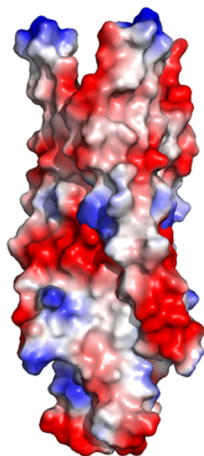

D

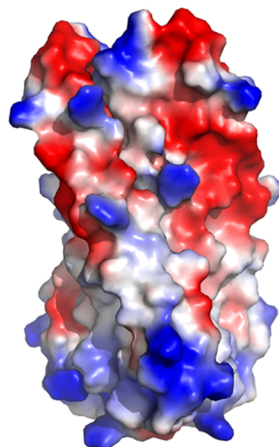

E

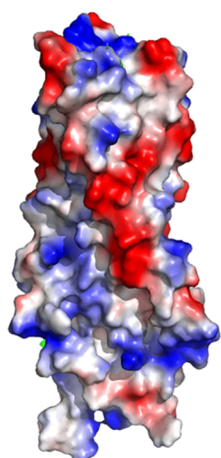

Fig. S1

Supplement: Additional file 1: Figure S1 — Supporting data for the EIAV gp45WT structure. (A) Superimposed structures of EIAV gp45WT (from Figure 1D) and the HIV CRF07 gp41 protein. The HIV CRF07 gp41 NHR and CHR domains are highlighted in magenta and pink colors, respectively, with a linker (yellow) between them. (B) Superimposed structures of EIAV gp45WT (from Figure 1D) and the SIV gp41 protein. The SIV gp41 NHR and CHR domains are highlighted in cyan and orange colors, respectively. (C-E) Surface charge potentials of the EIAV gp45, HIV CRF07 gp41, and SIV gp41. Negatively charged residues are colored in red and positively charged residues in blue. [file 1742-4690-11-26-S1.pdf]

A

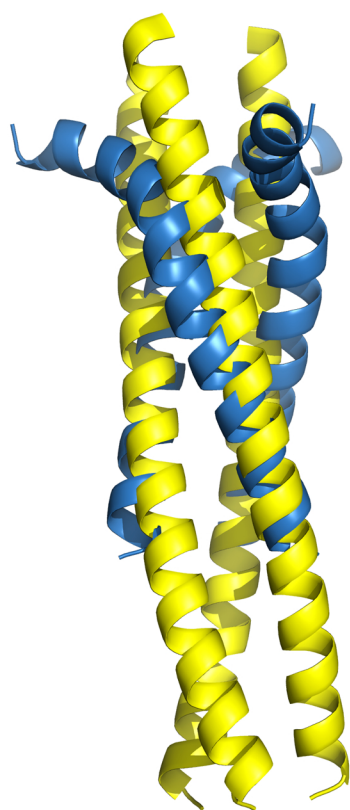

B

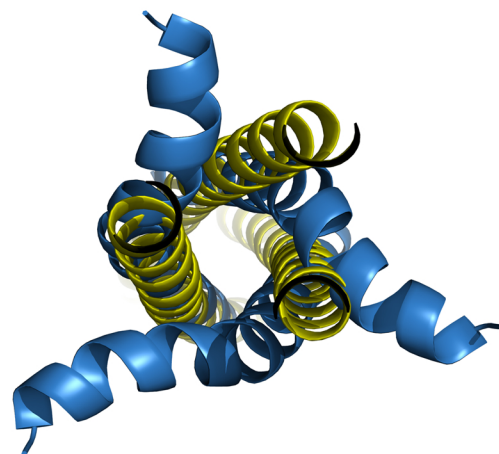

Fig. S2

Supplement: Additional file 2: Figure S2 — Superimposition of gp45VACCINE NHR and HIV gp41 NHR derived from crystal structure of Env trimer (PDB code 4NCO). Side-view for (A) and top-view for (B). gp45VACCINE NHR is shown as in Figure 2A and gp41 NHR is shown in blue. [file 1742-4690-11-26-S2.pdf]

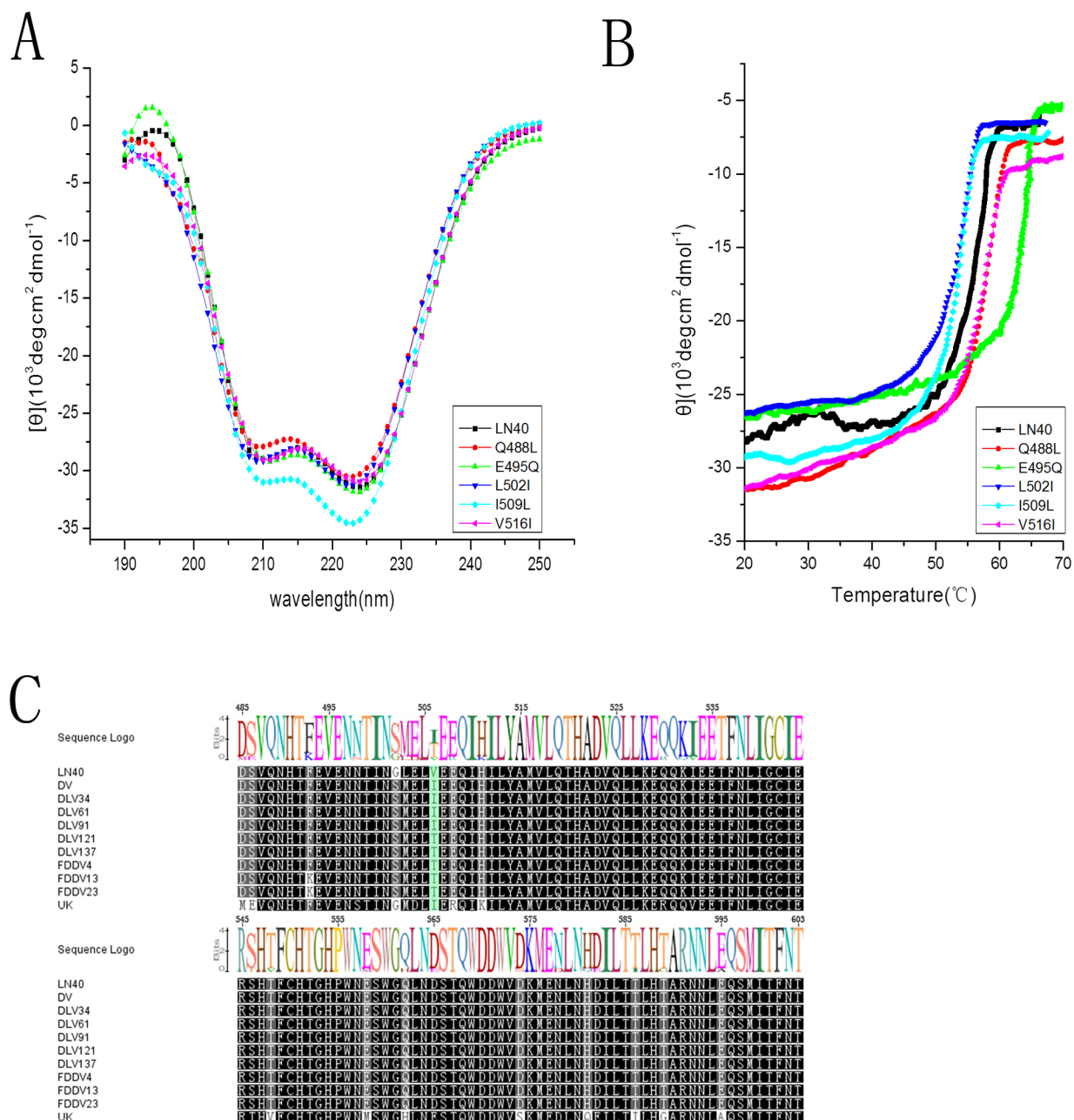

Fig. S3

Supplement: Additional file 3: Figure S3 — Supporting data for the EIAV gp45 mutant proteins. (A) Secondary structure representation of the EIAV gp45 mutants at position a, characterized by CD. (B) Thermostability results monitored by CD (at 222 nm) for the a mutants. (C) Sequence alignment of the EIAV gp45 ecto-domain. The LN40 represents the WT pathogenic strain isolated in Liaoning Province, China. The DV/DLV34/DLV61 series is classed as a pathogenic strain; the DLV121/DLV137/FDDV4/FDDV13/FDDV23 as non-pathogenic; with DLV91 depicted as the intermediate. The UK3 pathogenic strain is used in this study to construct the infectious clone. The specific residue at the 505 position is shown in green shadow. [file 1742-4690-11-26-S3.pdf]

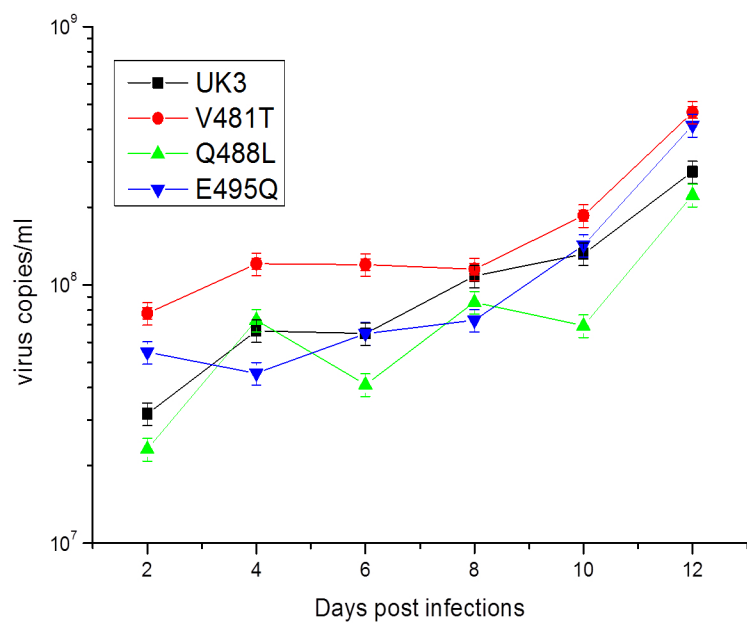

Fig. S4

Supplement: Additional file 4: Figure S4 — Replication analyses of the EIAV mutants at position a (as depicted in Figure 4A). [file 1742-4690-11-26-S4.pdf]
