# Supplementary material for: Structural and biochemical insights into the V/I505T mutation found in the EIAV gp45 vaccine strain
Source: Retrovirology. 2014 Mar 21;11:26. doi: 10.1186/1742-4690-11-26 (PMC3997929; doi:10.1186/1742-4690-11-26)
Supplement: Additional file 5: Table S1 — X-ray crystallographic data and refinement statistics for EIAV gp45. [file 1742-4690-11-26-S5.docx]

**Table S1**. X-ray crystallographic data and refinement statistics for EIAV gp45.

| **Crystals** | gp45_WT_ | gp45_VACCINE_ |
| --- | --- | --- |
| **Data collection** |  |  |
| Space group | *P6_3_* | *P6_3_* |
| Wavelength (Å) | 0.9792 | 0.9792 |
| Unit cell dimensions |  |  |
| a (Å) | 47.05 | 46.95 |
| b (Å) | 47.05 | 46.95 |
| c (Å) | 101.92 | 101.61 |
| α, β, γ (º) | 90, 90, 120 | 90, 90, 120 |
| Molecules per ASU^Ф^ | 1 | 1 |
| Resolution (Å) ^*^ | 1.9(1.93-1.90) | 2.0(2.03-2.00) |
| Completeness (%)^*^ | 99.4 (96.1) | 99.6(98.6) |
| Redundancy^*^ | 9.4 (5.0) | 9.1 (6.7) |
| No. of total reflections | 94335 | 78305 |
| No. of unique reflections | 10074 | 8643 |
| I/σ^*^ | 16.6 (2.9) | 11.1 (2.2) |
| R_sym_^*†^ | 4.5 (16.2) | 7.3 (46.1) |
| **Refinement statistics** |  |  |
| Resolution (Å) | 1.9 | 2.0 |
| No. of reflections | 10028 | 8604 |
| R_work_/R_free_ (%)^‡§^ | 17.83/22.32 | 18.51/23.20 |
| No. of atoms |  |  |
| Protein | 774 | 773 |
| Water | 180 | 68 |
| B-factors (Å^2^) |  |  |
| Protein | 20.61 | 35.20 |
| Water | 33.45 | 41.14 |
| R.m.s. deviations |  |  |
| Bond length (Å) | 0.006 | 0.007 |
| Bond angle (º) | 0.774 | 0.852 |
| **Ramachandran analysis** |  |  |
| Favored (%) | 100 | 100 |
| Allowed (%) | 0 | 0 |
| Outliers (%) | 0 | 0 |

ФASU=asymmetric unit.

*Values in parentheses are for the highest resolution shell.

†R_sym_ = Σ|I-<I>|/Σ<I>, where I is the observed intensity, and <I> is the average intensity of multiple observations of symmetry related reflections.

‡R = Σhkl||Fobs|-|Fcalc||/Σhkl|Fobs|

§R_free_ is calculated from 5% of the reflections excluded from refinement.
